# Predictors of inpatient outcomes of COVID‐19 infection in patients with cirrhosis in the early pandemic phase: A nationwide survey

**DOI:** 10.1002/jgh3.12998

**Published:** 2023-11-08

**Authors:** Abdullah Sohail, Khadija Naseem, Ahmad Khan, Talal Adhami, Kyle Brown

**Affiliations:** ^1^ Department of Internal Medicine University of Iowa Hospitals and Clinics Iowa City Iowa USA; ^2^ Cleveland Clinic Foundation Cleveland Ohio USA; ^3^ Department of Gastroenterology and Hepatology Case Western Reserve University Cleveland Ohio USA

**Keywords:** Advanced COVID‐19 therapies, chronic liver disease, inpatient mortality, National Inpatient Sample database, resource utilization

## Abstract

**Background and Aim:**

Previous studies conducted at single centers have suggested that patients with cirrhosis are at a greater risk for worse outcomes with COVID‐19. However, there is limited data on a national level in the United States. We aimed to study hospital‐related outcomes and identify the predictors of poor outcomes in patients with cirrhosis and concurrent COVID‐19.

**Methods:**

We queried 2020 National Inpatient and Readmission databases to identify all hospitalizations due to cirrhosis in adults with a diagnosis of COVID‐19. Primary outcomes included inpatient mortality, mechanical ventilation (MV), and intensive care unit (ICU) utilization. Secondary outcomes included mean length of stay (LOS) and mean hospitalization costs. We classified cirrhosis into compensated (CC) and decompensated (DC) groups.

**Results:**

We identified 25194 hospitalizations of adult patients due to cirrhosis with a concurrent diagnosis of COVID‐19. These patients had higher mortality (19.50% *vs* 6.19%, *P* ≤ 0.01), MV (11.7% *vs* 2.8%, *P* ≤ 0.01), ICU utilization (17.3% *vs* 8.1%, *P* ≤ 0.01), LOS (8.89 days *vs* 6.16 days, *P* ≤ 0.01), and total hospitalization costs ($24 817 *vs* $18 505, *P* ≤ 0.01) than those without COVID‐19. On subgroup analysis, patients in the DC group had higher mortality, LOS, and hospitalization costs compared to those in the CC group. On multivariate analysis, we also found that COVID‐19 infection, age, Charlson Comorbidity Index ≥3, acute kidney injury, end‐stage renal disease, septic shock, acute respiratory failure, MV, and ICU status were independent predictors for mortality.

**Conclusion:**

Our study suggests that COVID‐19 infection is an independent predictor of mortality in patients with cirrhosis, with threefold higher mortality and increased resource utilization. Early intervention through immunizations and advanced COVID‐19 therapies can help improve these outcomes.

## Graphical abstract

This study examines the impact of COVID‐19 on national‐level outcomes for patients with cirrhosis using 2020 National Inpatient Sample data. Patients with cirrhosis and COVID‐19 experience significantly higher mortality rates, increased resource utilization, longer hospital stays, and greater hospitalization costs compared to cirrhosis patients without COVID‐19. Decompensated cirrhosis patients face even worse outcomes, highlighting the importance of early intervention and advanced therapies to improve their prognosis.

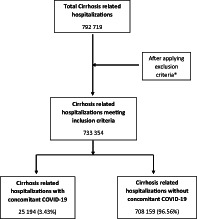

## Introduction

The coronavirus disease 2019 (COVID‐19) pandemic is an ongoing global health challenge that has triggered unprecedented direct and indirect medium‐ to long‐term effects on mortality and morbidity. According to the World Health Organization, there were more than 640 million confirmed cases as of December 2022, and more than 6 million people died because of the pandemic.^1^ Patients with chronic health conditions or already reduced life expectancies are considered the most vulnerable individuals. Although preventive health measures and immunizations have partially offset the risk of contraction and poor outcomes, patients with chronic diseases are still at an increased risk for poor outcomes.^2^ Among these patients, chronic liver disease (CLD), especially cirrhosis, is considered a high‐risk comorbid condition for severe COVID‐19 disease due to inherent immune dysregulation.^3^

Between 1999 and 2010, cirrhosis had a prevalence of 0.27% in the United States, with an all‐cause mortality rate of up to 26.4% per 2‐year interval in cirrhosis patients compared with 8.4% in propensity‐matched controls.^4^ Earlier studies during the pandemic showed that COVID‐19 is associated with significantly higher mortality and morbidity in patients with cirrhosis compared to the general population. Numerous studies have indicated that cirrhotic patients with COVID‐19 have a dire prognosis, with overall mortality rates as high as 35%.^5^, ^6^ In addition to the adverse inpatient outcomes, in some patients COVID‐19 symptoms tend to linger, causing repeated hospitalizations. Patients with cirrhosis, especially decompensated cirrhosis (DC), are a high‐risk population at baseline for readmissions, mainly due to the complications of portal hypertension.

Most previous studies on COVID‐19 in cirrhotic patients were from single centers or different healthcare systems, or had a small sample size. In addition, there is no nationwide study regarding the readmission rate and causes of hospital readmissions in patients with cirrhosis with concomitant COVID‐19 infection. A better understanding of the national data for inpatient outcomes and readmissions in patients with cirrhosis with COVID‐19 can aid in identifying areas for improvement and planning measures to improve patient care and minimize costs. We used the two largest databases to determine the inpatient outcomes, resource utilization, and 30‐day readmission rate in cirrhotic patients with COVID‐19.

## Methods

### Data source

We conducted a retrospective cohort study using the recently released National Inpatient Sample (NIS) 2020 and National Readmission Database (NRD) 2020 to identify all patients with cirrhosis and concomitant COVID‐19 infection. NIS and NRD are a part of the Healthcare Cost Utilization Project (HCUP), sponsored by the Agency of Healthcare Research and Quality (AHRQ).^7^ NIS and NRD are the largest publicly accessible databases in the United States, covering all payer healthcare information. The NIS 2020 contains data from 48 states covering 97% of the U.S. population.^8^ Similarly, the NRD 2020 includes data from 31 states covering 60.8% of all U.S. hospitalizations.^9^ The NIS database provides estimates of in‐hospital mortality and resource utilization associated with various conditions to improve the quality and outcomes of in‐hospital care. In comparison, the NRD provides national‐level estimates of in‐hospital readmissions in the United States. Both are designed in a stratified probability sample representing 20% of all non‐federal acute care inpatient hospitalizations. The stratification follows the American Hospital Association criteria and is based on the U.S. census region, division, hospital control, location, teaching status, and hospital bed size.^7^ Both databases contain patient‐level and hospital‐level information. Hospital‐level data include control/ownership, teaching/non‐teaching status, number of beds, hospital region (not in NRD), and rural/urban location. Patient‐level data contain demographics including age, gender, race (not in NRD), median income by zip quartile, principal and secondary diagnoses at discharge, the total number of patients who died, length of stay (LOS), procedures performed, and total hospitalization cost and charges. The discharge diagnosis and procedure codes are identified using the *International Classification of Diseases, Tenth Revision, and Clinical Modification* (ICD10‐CM). NIS is de‐identified data, but NRD assigns a unique database identification number for each patient.^8^ This unique number can be used to identify all admissions for each patient. A readmission was defined as any non‐traumatic admission within 30 days of the index admission. If the patient had multiple readmissions, we considered only the first readmission. All patients who died during the index hospitalization were excluded from the readmission study. In our study, the NIS data were used to estimate inpatient mortality and resource utilization while NRD was used to assess the 30‐day readmission rate.

### Ethical statement

Both NIS and NRD datasets are de‐identified and do not directly involve “human subjects,” as defined by federal regulations and guidance (https://www.hhs.gov/ohrp/regulations‐and‐policy/regulations/common‐rule/index.html). Therefore, NIS/NRD do not require institutional review board (IRB) approval. All procedures performed were in accordance with the ethical standards of the 1964 Helsinki Declaration and its later amendments or comparable ethical standards.

### Study population

We identified all hospitalization related to cirrhosis and concomitant COVID‐19 infection using ICD‐10 codes (provided in Supplementary Data). We also performed subgroup analysis to categorize cirrhosis into compensated (CC) and decompensated cirrhosis (DC) groups based on the presence of one or more diagnosis codes for clinically significant portal hypertension‐related complications. We excluded patients under 18 years of age and those with elective admissions. To arrive at an accurate readmission rate, we excluded all the index hospitalizations in December because NRD keeps track of hospitalizations on a calendar year basis (i.e. January 1 to December 31) without linkage to the previous or following year.

### Study outcome

The primary outcomes of our study were inpatient mortality, mechanical ventilation (MV), acute respiratory failure (ARF), intensive care utilization (ICU) requirement, and the all‐cause 30‐day readmission rate, with the most common reason leading to these readmissions. The secondary outcomes included healthcare utilization, including the LOS and the total hospitalization charges in both groups. We also conducted univariate and multivariate regression analyses to identify the independent predictors of mortality, LOS, and hospitalization cost.

### Patient and hospital characteristics

We used NIS and NRD variables to identify the patient's age, gender, median household income for the patient's zip code, primary insurance payer (Medicare, Medicaid, private insurance, self‐pay, or uninsured), patient residential area (a large metropolitan area with 1 million residents, a small metropolitan area with less than 1 million residents, micropolitan areas, and not metropolitan/micropolitan). Hospital characteristics, including size (large, medium, or small), location (urban or rural), and teaching status (teaching or non‐teaching) were also assessed. The ICD‐10 codes were used to identify comorbid conditions, including hypertension, acute kidney injury (AKI), chronic kidney disease (CKD), end‐stage renal disease (ESRD), diabetes mellitus (DM), acute respiratory failure (ARF), chronic pulmonary disease (CPD), and MV (Supplemental Files). NIS also provides information about the total hospitalization charges the hospital billed for each hospitalization. The Charlson Comorbidity Index (CCI) was used to assess the burden of comorbidities in both groups of patients. In studies utilizing administrative databases, the CCI score predicts 1‐year survival in patients based on the presence or absence of multiple comorbidities.^10^

### Statistical analysis

All statistical analyses were conducted using STATA, version 16.0 (Stata Corp, College Station, Texas, USA). To analyze descriptive statistics for patients and hospital characteristics, Pearson's chi‐square test was used for categorical variables and Student's *t*‐test for continuous variables. Continuous variables are described as means with standard deviation (SD), whereas categorical variables are reported as proportions or percentages. We used case‐by‐case deletion to remove missing data, and less than 5% of the data for LOS, hospitalization cost, and mortality were missing. An α‐value of less than 0.05 was chosen as the level of significance. To assess the effect of COVID‐19 on secondary outcomes, we used bivariate linear and logistic regression analyses, followed by multivariate regression models to account for confounding factors. We chose variables for the multivariate models with a significance level of 0.2 in the bivariate analysis. All observations with missing values were excluded from the study. The total hospitalization cost variable is not available in NIS as a default; hence we used the HCUP cost‐to‐charge ratio.^11^ The survival analysis was performed, with the time from the discharge to readmission considered a time variable and death as a failure. Patients were censored if they were alive on their 30th day of discharge.

## Results

### Baseline characteristics

**Figure 1:**
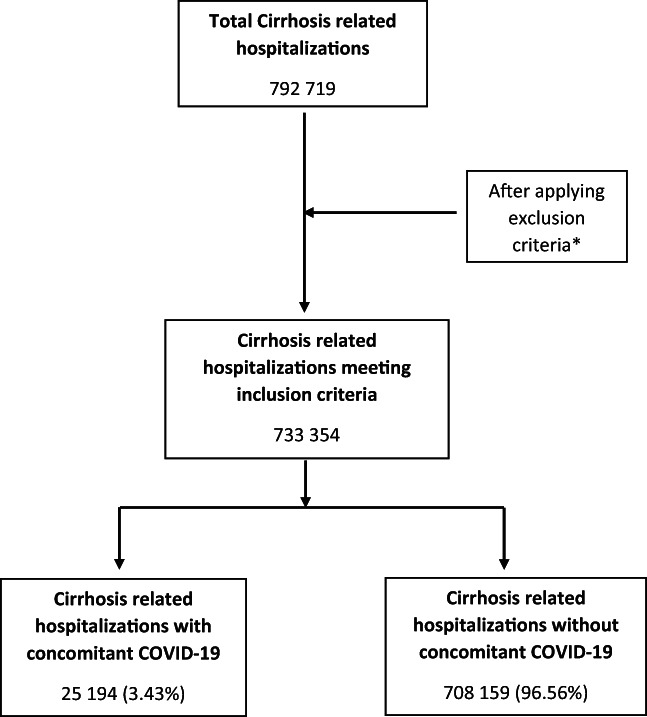
Patient selection flow diagram. *Non‐adult patients, elective admissions, patients with missing data related to outcomes.

We identified a total of 733 354 cirrhosis‐related hospitalizations in adult patients, out of which 25 194 had a concurrent diagnosis of COVID‐19 infection (Fig. 1). In the cirrhosis + COVID group, 12 745 (50.59%) met the definition of DC compared to 12 449 (49.41%) for CC. The cirrhosis + COVID group patients were older, with a mean age of 62.20 years *versus* 60.27 years for those with cirrhosis but without COVID‐19 (*P* < 0.01). A significantly larger proportion of the patients in the cirrhosis + COVID group were older than 65 years compared to those with cirrhosis without COVID‐19 infection (49.2% with COVID *vs* 36% without COVID‐19, *P* < 0.01). In both groups, the majority of patients were females (52.1% with COVID‐19 *vs* 55.2% without COVID‐19; *P* < 0.01) and White (73.6% with COVID‐19 *vs* 77.8% without COVID‐19; *P* < 0.01). More than half of the patients with both cirrhosis and COVID‐19 (55.4%) had Medicare as their primary payer; this proportion was smaller among the patients with cirrhosis without COVID‐19 (46%; *P* < 0.01). In terms of individual comorbidities, more patients had a CCI > 3 (29.3% with COVID‐19 *vs* 26.7% without COVID‐19; *P* < 0.01) and CPD (24.7% with COVID‐19 *vs* 22.4% without COVID‐19; *P* < 0.01) in the COVID group compared to the non‐COVID group; however, ESRD was significantly higher in the non‐COVID group (6.9% with COVID‐19 *vs* 9.1% without COVID‐19; *P* < 0.01). A larger number of the patients in the cirrhosis + COVID group were discharged to a skilled nursing facility compared to the non‐COVID group (18.7% with COVID‐19 *vs* 11.2% without COVID‐19, *P* < 0.01). There were no statistically significant differences in median income between the two groups of patients based on zip code. The remaining patient and hospital characteristics were comparable between the two groups (Table 1).

**Table 1:** Baseline patient and hospital characteristics for hospitalized cases of cirrhosis with and without COVID‐19 infection

Patient characteristics	Cirrhosis with COVID‐19, *N* (%)	Cirrhosis without COVID‐19, *N* (%)	*P*‐value
No. of patients	25 194 (3.43%)	708 159 (96.56%)	
Female	13 126 (52.1%)	390 903 (55.2%)	<0.01
Mean age (years)	62.20	60.27	
Age distribution (years)			<0.01
18–34	2368 (9.4%)	132 426 (18.7%)	
35–44	2293 (9.1%)	99 850 (14.1%)	
45–64	8138 (32.3)	221 654 (31.3%)	
>65	12 395 (49.2%)	254 937 (36%)	
Race			<0.01
White	18 542 (73.6%)	550 947 (77.8%)	
African American	3224 (12.8%)	84 270 (11.9%)	
Hispanic	2494 (9.9%)	46 030 (6.5%)	
Other	932 (3.7%)	26 910 (3.8%)	
Charlson Comorbidity Index			<0.01
0	7784 (30.9%)	276 182 (39%)	
1	5819 (23.1%)	145 172 (20.5%)	
2	4207 (16.7%)	97 017 (13.7%)	
3 or more	7306 (29.3%)	189 078 (26.7%)	
Comorbidities			
Hypertension	50 (0.2%)	1416 (0.2%)	0.82
Diabetes mellitus	152 (0.6%)	4248 (0.6%)	0.97
Chronic kidney disease			<0.01
Stage 2	252 (1%)	9206 (1.3%)	
Stage 3	1990 (7.9%)	36 116 (5.1%)	
Stage 4	705 (2.8%)	19 828 (2.8%)	
Stage 5	76 (0.3%)	2124 (0.3%)	
Unspecified	1285 (5.1%)	35 408 (5%)	
End‐stage renal disease	1738 (6.9%)	64 442 (9.1%)	<0.01
Chronic pulmonary disease	6223 (24.7%)	158 628 (22.4%)	<0.01
Congestive heart failure	6299 (25%)	183 413 (25.9%)	0.17
Median income based on zip codes			0.11
$1–$38 999	6953 (27.6%)	177 747 (25.10%)	
$39 000–$47 999	6676 (26.5%)	190 494 (26.9%)	
$48 000–$62 999	5819 (23.1%)	174 207 (24.6%)	
>$63 000	5744 (22.8%)	166 417 (23.5%)	
Insurance provider			<0.01
Medicare	13 957 (55.4%)	325 753 (46%)	
Medicaid	2620 (10.4%)	114 721 (16.2%)	
Private	8137 (32.3%)	239 357 (33.8%)	
Self‐pay	503 (2%)	28 326 (4%)	
Patient disposition			<0.01
Home	12 824 (50.9%)	471 633 (66.6%)	
Home with home health	3779 (15%)	111 180 (15.7%)	
Skilled nursing facility	4711 (18.7%)	79 313 (11.2%)	
Against medical advice	328 (1.3%)	17 703 (2.5%)	
Hospital characteristics			
Hospital teaching status			0.1
Non‐teaching	6600 (26.2%)	173 498 (24.5%)	
Teaching	18 593 (73.8%)	534 660 (75.5%)	
Hospital bed size			0.42
Small	5769 (22.9%)	158 627 (22.4%)	
Medium	6575 (26.1%)	195 451 (27.6%)	
Large	12 848 (51%)	354 079 (50%)	
Hospital region			<0.01
Northeast	5265 (20.9%)	151 546 (21.4%)	
Midwest	6928 (27.5%)	165 709 (23.4%)	
South	9246 (36.7%)	270 516 (38.2%)	
West	3779 (15%)	120 387 (17%)	

### Primary outcomes

Patients with cirrhosis and COVID‐19 were found to have higher all‐cause mortality (19.50% with COVID‐19 *vs* 6.19% without COVID‐19, *P* < 0.01) with an increased MV requirement (11.7% with COVID‐19 *vs* 2.8% without COVID‐19, *P* < 0.01) and ICU utilization (17.3% with COVID‐19 *vs* 8.1% without COVID‐19, *P* < 0.01) as compared to cirrhotic patients without COVID‐19 infection (Table 2). In the subgroup analysis, patients with DC and COVID‐19 had an even higher mortality as compared to the CC group with COVID‐19 (23.04% with DC *vs* 16.04% with CC, *P* < 0.01). On multivariate analysis, we also found that COVID‐19 infection (adjusted OR [adj OR] = 2.63; confidence interval [CI] = 2.38–2.90; *P* < 0.01), age (adj OR = 1.03; CI = 1.03–1.06; *P* < 0.01), CCI ≥ 3 (adj OR = 1.5; CI = 1.35–1.7; *P* < 0.01), AKI (adj OR = 2.66; CI = 2.5–2.83; *P* < 0.01), ESRD (adj OR = 1.57; CI = 1.43–1.73; *P* < 0.01), septic shock (adj OR = 3.85; CI = 3.56–4.16; *P* < 0.01), ARF (adj OR = 3.28; CI = 3.06–3.51; *P* < 0.01), MV (adj OR = 1.94; CI = 1.67–2.26; *P* < 0.01), and ICU (adj OR = 3.38; CI = 2.91–3.93; *P* < 0.01) were independent predictors for mortality after adjusting for potential confounders (Table 3). The 30‐day readmission rate in patients with cirrhosis was 8.28% in COVID‐19 *versus* 17.54% without COVID‐19. Sepsis was the most common cause of readmissions in the cirrhosis + COVID group, followed by alcoholic cirrhosis of the liver and hepatic failure, unspecified without coma.

**Table 2:** Outcomes for hospitalized cases of cirrhosis with and without COVID‐19 infection

Outcomes			
Patient Characteristics	Cirrhosis with COVID‐19, *N* (%)	Cirrhosis without COVID‐19, *N* (%)	*P*‐value
Mortality	4914 (19.50%)	43 879 (6.19%)	<0.01
Mechanical ventilation	2947 (11.7%)	19 828 (2.8%)	<0.01
Intensive care unit	4358 (17.3%)	57 360 (8.1%)	<0.01
Acute respiratory failure	11 060 (43.9%)	106 932 (15.1%)	<0.01
Septic shock	2897 (11.5%)	37 532 (5.3%)	<0.01
Mean length of stay (days)	8.89	6.16	<0.01
Mean hospitalization cost	$24 817	$18 505	<0.01

**Table 3:** Bivariate and multivariate logistic regression showing predictors for mortality in patients with cirrhosis hospitalized with COVID‐19 infection

		Bivariate logistic regression			Multivariate logistic regression	
	Odds ratio	95% confidence interval	*P*‐value	Odds ratio	95% confidence interval	*P*‐value
COVID‐19 Infection	3.71	(3.45–4.00)	<0.01	2.63	(2.38–2.90)	<0.01
Female	0.93	(0.89–0.96)	<0.01	0.93	(0.88–0.98)	<0.01
Race						
White			Reference			
Black	1.18	(1.10–1.27)	<0.01	1.04	(0.95–1.14)	0.364
Hispanic	1.02	(0.96–1.09)	0.48	0.85	(0.79–0.93)	<0.01
Other	1.31	(1.22–1.43)	<0.01	1.03	(0.92–1.14)	0.635
Age (mean)	1.01	(1.00–1.01)	<0.01	1.03	(1.03–1.06)	<0.01
18–34			Reference			
35–44	1.3	(1.11–1.52)	<0.01	0.93	(0.76–1.14)	0.5
45–64	1.43	(1.24–1.65)	<0.01	0.69	(0.56–0.86)	<0.01
≥65	1.7	(1.48–1.96)	<0.01	0.68	(0.52–0.89)	<0.01
Charlson Comorbidity index						
1			Reference			
2	1.16	(1.04–1.31)	<0.01	1.01	(0.87–1.16)	0.935
3 or more	2.21	(2.02–2.41)	<0.01	1.51	(1.35–1.7)	<0.01
Median income based on the zip code						
$1–$38 999			Reference			
$39 000–$47 999	0.96	(0.91–1.02)	0.21	0.95	(0.88–1.02)	0.144
$48 000–$62 999	0.95	(0.90–1.01)	0.13	0.9	(0.83–0.97)	<0.01
>$63 000	0.96	(0.89–1.03)	0.89	0.86	(0.78–0.93)	<0.01
Insurance provider						
Medicare			Reference			
Medicaid	0.97	(0.92–1.02)	0.26	1.23	(1.13–1.34)	<0.01
Private	1.05	(0.99–1.11)	0.1	1.26	(1.16–1.38)	<0.01
Uninsured	1	(0.90–1.10)	0.93	1.71	(1.5–1.96)	<0.01
Comorbidities						
Acute Kidney Injury	5.73	(5.46–6.02)	<0.01	2.66	(2.5–2.83)	<0.01
Chronic kidney disease	1.03	(1.03–1.05)	<0.01	0.96	(0.94–0.98)	<0.01
ESRD	1.53	(1.43–1.64)	<0.01	1.57	(1.43–1.73)	<0.01
Congestive heart failure	1.26	(1.20–1.32)	<0.01	0.84	(0.79–0.89)	<0.01
Hospital teaching status						
Non‐teaching			Reference			
Teaching	1.16	(1.10–1.23)	<0.01	0.97	(0.9–1.05)	0.524
Hospital size						
Small			Reference			
Medium	1.18	(1.10–1.28)	<0.01	1.07	(0.97–1.18)	0.173
Large	1.21	(1.13–1.30)	<0.01	1.05	(0.96–1.15)	0.331
Hospital region						
Northeast			Reference			
Midwest	0.86	(0.79–0.94)	<0.01	0.66	(0.6–0.74)	<0.01
South	0.88	(0.81–0.95)	0.01	0.75	(0.67–0.83)	<0.01
West	1.08	(0.99–1.17)	<0.01	0.96	(0.87–1.07)	0.486
Complications						
Intensive care unit	23.64	(22.44–24.90)	<0.01	3.38	(2.91–3.93)	<0.01
Mechanical ventilation	24.02	(22.84–25.27)	<0.01	1.94	(1.67–2.26)	<0.01
Acute respiratory failure	12.02	(11.46–12.59)	<0.01	3.28	(3.06–3.51)	<0.01
Septic shock	19.89	(18.80–21.02)	<0.01	3.85	(3.56–4.16)	<0.01

### Secondary outcomes and resource utilization

The patients in the cirrhosis + COVID group had higher mean LOS (8.89 days with COVID‐19 *vs* 6.16 days without COVID‐19, *P* < 0.01) and mean total hospitalization cost ($24 817 with COVID‐19 *vs* $18 505 without COVID‐19, *P* < 0.01) compared to patients with cirrhosis but without COVID‐19. On subgroup analysis, the DC group with COVID‐19 had even higher mean LOS (9.86 days *vs* 7.93 days; *P* < 0.01) and total hospitalization cost ($28 476 *vs* $21 218; *P* < 0.01) than the CC Group with COVID‐19. COVID‐19 infection was also independently associated with increased LOS (β = 1.66; CI = 1.42–1.91; *P* < 0.01) and hospitalization cost (β = 1002; CI = 46–1957; *P* = 0.04).

AKI (β = 1.76, CI = 1.64–1.89; *P* < 0.01), ESRD (*β* = 1.02, CI = 0.83–1.22; *P* < 0.01), ICU (*β* = 2.42; CI = 1.67–2.81; *P* < 0.01), ARF (*β* = 2.02; CI = 1.87–2.18; *P* < 0.01), and septic shock (*β* = 1.33; CI = 1.03–1.64; *P* < 0.01) were found to be independent predictors for increased LOS (Table 4). Moreover, CCI > 3 (*β* = 3520; CI = 3093–3947; *P* < 0.01), AKI (*β* = 5755; CI = 5104–6405; *P* < 0.01), ESRD (*β* = 5942; CI = 4853–7032; *P* < 0.01), ICU (*β* = 17 745; CI = 13 566–21 925; *P* < 0.01), ARF (*β* = 6698; CI = 5952–74 452; *P* < 0.01), and septic shock (*β* = 9248; CI = 7542–10 954; *P* < 0.01) were independent predictors of the hospitalization costs (Table 5).

**Table 4:** Bivariate and multivariate logistic regression showing predictors for length of stay (LOS) in patients with cirrhosis hospitalized with COVID‐19 infection

		Bivariate logistic regression			Multivariate logistic regression	
	Coefficient	95% confidence interval	*P*‐value	Coefficient	95% confidence interval	*P*‐value
COVID‐19 infection	2.78	(2.53–3.03)	<0.01	1.66	(1.42–1.91)	<0.01
Female	−0.11	(−0.18 to 0.03)	0.006	0	(−0.07–0.07)	0.997
Race						
White			Reference			
Black	0.77	(0.6–0.93)	<0.01	0.25	(0.09–0.41)	0.003
Hispanic	0.05	(−0.12–0.22)	0.566	−0.14	(−0.29–0.02)	0.084
Other	0.25	(0.05–0.45)	0.016	−0.06	(−0.24–0.12)	0.492
Age (mean)	0.00	(−0.01–0.00)	<0.01	0.00	(−0.01–0.00)	<0.01
18–34			Reference			
35–44	−0.37	(−0.66 to 0.09)	0.009	−0.4	(−0.67 to 0.13)	0.004
45–64	−0.24	(−0.49–0.01)	0.056	−0.22	(−0.49–0.06)	0.119
≥65	−0.35	(−0.61 to −0.08)	0.01	−0.08	(−0.42–0.26)	0.646
Charlson Comorbidity index						
1			Reference			
2	0.4	(0.24–0.56)	<0.01	0.22	(0.07–0.37)	0.004
3 or more	1.55	(1.42–1.67)	<0.01	0.71	(0.58–0.83)	<0.01
Median income based on the zip code						
$1–$38 999			Reference			
$39 000–$47 999	−0.11	(−0.22–0)	0.049	0.03	(−0.08–0.13)	0.613
$48 000–$62 999	−0.04	(−0.16–0.08)	0.512	0.07	(−0.04–0.19)	0.228
>$63 000	0.01	(−0.15–0.17)	0.882	0.07	(−0.07–0.21)	0.331
Insurance provider						
Medicare			Reference			
Medicaid	0.57	(0.45–0.69)	<0.01	0.64	(0.5–0.77)	<0.01
Private	0.08	(−0.04–0.21)	0.177	0.1	(−0.02–0.22)	0.103
Uninsured	−0.45	(−0.65 to 0.26)	<0.01	−0.06	(−0.26–0.14)	0.567
Comorbidities						
Diabetes mellitus	1.01	(0.43–1.58)	<0.01	0.4	(−0.2–1.01)	0.192
Hypertension	1.76	(0.95–2.58)	<0.01	1.45	(0.63–2.27)	<0.01
Acute kidney injury	2.95	(2.81–3.08)	<0.01	1.77	(1.64–1.89)	<0.01
Chronic kidney disease	0.07	(0.05–0.09)	<0.01	−0.12	(−0.14 to 0.09)	<0.01
ESRD	1.47	(1.27–1.67)	<0.01	1.02	(0.83–1.22)	<0.01
Congestive heart failure	1.08	(0.99–1.17)	<0.01	0.52	(0.43–0.62)	<0.01
Hospital teaching status						
Non‐teaching			Reference			
Teaching	1.11	(0.98–1.24)	<0.01	0.84	(0.72–0.95)	<0.01
Hospital size						
Small			Reference			
Medium	0.46	(0.31–0.62)	<0.01	0.35	(0.21–0.49)	<0.01
Large	1.13	(0.97–1.29)	<0.01	1.05	(0.9–1.2)	<0.01
Hospital region						
Northeast			Reference			
Midwest	−0.57	(−0.83 to 0.3)	<0.01	−0.84	(−1.06 to 0.62)	<0.01
South	−0.65	(−0.89 to 0.41)	<0.01	−0.58	(−0.79 to 0.38)	<0.01
West	−0.76	(−1.02 to 0.51)	<0.01	−1.06	(−1.29 to 0.83)	<0.01
Complications						
Septic shock	5.24	(4.94–5.53)	<0.01	1.33	(1.03–1.64)	<0.01
Intensive care unit	5.95	(5.67–6.23)	<0.01	2.24	(1.67–2.81)	<0.01
Acute respiratory failure	4.29	(4.11–4.47)	<0.01	2.02	(1.87–2.18)	<0.01
Mechanical ventilation	6.11	(5.82–6.39)	<0.01	0.65	(0.07–1.23)	0.02

**Table 5:** Bivariate and multivariate logistic regression showing predictors for hospitalization costs in patients with cirrhosis hospitalized with COVID‐19 infection

		Bivariate logistic regression			Multivariate logistic regression	
	Coefficient	95% confidence interval	*P*‐value	Coefficient	95% confidence interval	*P*‐value
COVID‐19 infection	6312	(5347–7277)	<0.01	1002	(46–1957)	0.04
Female	−912	(−1176 to 649)	<0.01	−295	(−554 to 36)	0.026
Race						
White			Reference			
Black	1780	(1119–2441)	<0.01	810	(234–1386)	0.006
Hispanic	3522	(2529–4515)	<0.01	1584	(832–2337)	<0.01
Other	5134	(3828–6439)	<0.01	1990	(886–3093)	<0.01
Age (mean)	−69.50	(−87.44–51.57)	<0.01	−87.87	(−112.33 to −63.40)	<0.01
18–34			Reference			
35–44	−3226	(−4687 to 1765)	<0.01	−2330	(−3623 to 1037)	<0.01
45–64	−3639	(−4980 to 2298)	<0.01	−1586	(−2897 to 274)	0.018
≥65	−4649	(−6152 to 3147)	<0.01	−861	(−2449 to −728)	0.288
Charlson Comorbidity index						
1			Reference			
2	1446	(995–1896)	<0.01	1585	(1160–2011)	<0.01
3 or more	6592	(6088–7096)	<0.01	3520	(3093–3947)	<0.01
Median income based on the zip code						
$1–$38 999			Reference			
$39 000–$47 999	811	(365–1257)	<0.01	845	(437–1253)	<0.01
$48 000–$62 999	2433	(1838–3028)	<0.01	1797	(1264–2330)	<0.01
>$63 000	5333	(4385–6280)	<0.01	4010	(3227–4793)	<0.01
Insurance provider						
Medicare			Reference			
Medicaid	2194	(1696–2692)	<0.01	713	(265–1161)	0.002
Private	2994	(2294–3695)	<0.01	2060	(1460–2660)	<0.01
Uninsured	−2770	(−3490 to 2050)	<0.01	−703	(−1399 to 7)	0.048
Comorbidities						
Diabetes mellitus	1802	(339–3265)	0.016	−524	(−1900–851)	0.455
Hypertension	2888	(−752–6528)	0.12	2613	(−1046–6272)	0.162
Acute kidney injury	11 771	(10939–12 603)	<0.01	5755	(5104–6405)	<0.01
ESRD	7554	(6426–8681)	<0.01	5942	(4853–7032)	<0.01
Congestive heart failure	2303	(1875–2731)	<0.01	412	(−45–868)	0.077
COPD	−1144	(−1582 to 706)	<0.01	−839	(−1161 to 516)	<0.01
Hospital teaching status						
Non‐teaching			Reference			
Teaching	4955	(4202–5708)	<0.01	3306	(2666–3947)	<0.01
Hospital size						
Small			Reference			
Medium	1611	(843–2379)	<0.01	1264	(577–1951)	<0.01
Large	5513	(4515–6511)	<0.01	4705	(3796–5615)	<0.01
Hospital region						
Northeast			Reference			
Midwest	−3856	(−5527 to 2186)	<0.01	−4337	(−5783 to 2890)	<0.01
South	−5805	(−7357 to 4253)	<0.01	−4695	(−6038 to 3352)	<0.01
West	2042	(141–3943)	0.035	660	(−1083–2404)	0.458
Complications						
Septic shock	29 181	(27450–30 912)	<0.01	9248	(7542–10 954)	<0.01
Intensive care unit	34 850	(33179–36 521)	<0.01	17 745	(13566–21 925)	<0.01
Acute respiratory failure	19 306	(18406–20 206)	<0.01	6698	(5952–7445)	<0.01
Mechanical ventilation	35 464	(33912–37 015)	<0.01	6286	(2404–10 168)	<0.01

## Discussion

To our knowledge, this is the first study in the United States to present hospital‐related outcomes of COVID‐19 infection in patients with cirrhosis at the national level. Our study findings suggest that the inpatient mortality rate of patients with cirrhosis and COVID‐19 infection, particularly those with clinically significant portal hypertension, is 3 times higher than patients with cirrhosis without COVID‐19. In addition, COVID‐19 infection is an independent predictor of mortality in these patients after controlling for confounding factors. Moreover, advanced age, CCI > 3, AKI, ESRD, ICU care or MV requirement, and development of septic shock and respiratory failure were independent predictors of mortality (Table 3). Patients with cirrhosis are also more likely to develop severe disease with an increased requirement for MV and intensive care utilization with COVID‐19 infection, which may contribute to increased LOS and hospitalization charges. However, patients with cirrhosis and concurrent COVID‐19 infection have a lower all‐cause 30‐day readmission rate than those without COVID‐19, which may be explained by the higher inpatient mortality in patients with COVID‐19 infection during index hospitalizations.

Although the exact mechanism is still unclear, excess systemic inflammation, intestinal dysbiosis, cirrhosis‐induced immunological dysfunction, and coagulopathies may play a role in the etiology of cirrhosis patients with a greater risk of mortality and severity following COVID‐19 infection.^12^ Earlier studies conducted during the pandemic focused on the outcomes of COVID‐19 in patients with chronic liver disease and, based on subgroup analysis, reported mortality rates as high as 40% in patients with cirrhosis.^13^, ^14^, ^15^ However, most of these studies were conducted in single centers, had a small sample size from various healthcare systems, and did not distinguish the differences in outcomes between CC and DC.^13^, ^14^ Our study period corresponds to the earlier phase of the pandemic (2020) and reports relatively low inpatient mortality compared to some earlier studies. Moreover, on subgroup analysis, patients with DC had higher mortality with COVID‐19 than those with CC. Our study design has several unique features that improve the generalizability of our findings and advance our understanding of COVID‐19 disease in cirrhosis. These features include a subgroup analysis of cirrhosis, a broad representation of demographics, and a large nationwide sample size. On multivariate analysis among patients with cirrhosis, COVID‐19 infection was associated with a 2.63 times higher risk of in‐hospital mortality. A recent meta‐analysis analyzing 40 studies by Nagarajan *et al*. found that patients with cirrhosis and concurrent COVID‐19 had 3 times higher odds of mortality than those without cirrhosis, which is consistent with our study findings.^15^ However, most of the studies included in that meta‐analysis were conducted outside the United States, representing different patient populations with varying access to various healthcare systems. In contrast, our study's results represent the U.S. population at a national level regardless of hospital size, academic status, rural/urban location, and geographical region.

To gain a better understanding of the risk factors and patterns associated with adverse COVID‐19 infection outcomes, we conducted a multivariate analysis to find the predictors of inpatient mortality. Earlier, some studies had also reported that certain demographic factors were associated with poor outcomes in cirrhosis and COVID‐19. These included increased age, male gender, and ethnicity, primarily Black and Hispanic populations.^16^, ^17^ However, we found that COVID‐19 infection, advanced age, and CCI ≥ 3 were major cofactors in raising the mortality risk of patients with cirrhosis. Consistent with previous research, we found that female gender was associated with a lower risk of death in patients with cirrhosis and COVID‐19.^17^ Furthermore, in the COVID group, other comorbid conditions and inpatient complications, such as AKI, ESRD, ARF, MV, and ICU status, were independent predictors of mortality.

To date, our study is the only nationwide estimate of the 30‐day readmission rate and its causes among patients with cirrhosis and COVID‐19 infection. Using NRD, the 30‐day readmission rate among patients with cirrhosis and COVID‐19 infection was 8.28%, which is lower than the rate of 17.54% among patients with cirrhosis without COVID‐19 infection. The higher death rate during the initial hospital stay may have contributed to a lower readmission rate among patients with COVID‐19. Although there is no previous data on the readmission rate and causes in patients with cirrhosis and concurrent COVID‐19 infection, Garg *et al*. conducted a retrospective analysis to identify the most common causes of readmissions in patients with cirrhosis alone.^18^ They reported that the three most common causes of readmissions among these patients were liver diseases–not otherwise specified, substance abuse, and hepatitis, with only a small percentage of readmitted patients being diagnosed with sepsis. In contrast, our findings suggest that sepsis was the most common reason for readmissions among patients with cirrhosis and COVID‐19 infection, indicating that these patients may be more susceptible to infections after discharge, resulting in repeat hospitalizations. Patients with cirrhosis have worse outcomes with sepsis and septic shock, and some studies report mortality rates as high as 75%.^19^, ^20^ Our study results should encourage clinicians to implement infection control protocols and closely monitor these patients for signs of infection after discharge. Additionally, further research is needed to understand the underlying factors that increase the susceptibility of these patients to infections.

In terms of resource utilization, when compared to the non‐COVID group, patients in the COVID group, especially those with DC, had higher mean LOS and mean total hospitalization costs. Moreover, a more significant proportion of the patients in the COVID group were discharged to a skilled nursing facility compared to the non‐COVID group (Table 1). From 2001 to 2011, the annual cost of cirrhosis‐related hospitalizations in the United States rose more than twofold, from $4.8 billion to $9.8 billion.^21^ Inpatient hospitalization and readmissions significantly increase healthcare resource utilization among patients with cirrhosis.^22^ Hirode *et al*. found that from 2012 to 2016, the average inflation‐adjusted cost of cirrhosis‐related hospitalizations in the United States increased from $16 285 to $19 185 per admission.^23^ Our results show a significantly higher mean hospitalization cost for patients with cirrhosis and COVID‐19 ($24 817), which could be explained by the increased utilization of MV, ICU, and increased LOS.^24^ To the best of our knowledge, our study is unique in providing data on the resource utilization of patients with cirrhosis and COVID‐19 infection in the United States.

We acknowledge certain limitations to our study. Most of these limitations stem from using a claims‐based administrative database. These databases utilize ICD‐10 codes for diagnosis and procedure documentation, which are prone to under‐ or over‐reporting erroneously. Our study includes only the inpatient data and does not include information on the outcomes of outpatients with cirrhosis who had non‐severe COVID‐19. Since this database does not have laboratory data, we were unable to assess the severity of illness based on the Model End‐Stage Liver Disease—Sodium scores or Child–Turcotte–Pugh Class. Similarly, the database lacks the necessary granularity to perform certain detailed analyses. We could not determine the patient's liver transplant status. This database also lacks pharmacological information, limiting our ability to assess the efficacy of specific therapies. Finally, we could not capture out‐of‐state readmissions because NRD is derived from state‐based databases and does not use the exact linkage identification between states. We assume, however, that a limited number of out‐of‐state patients were readmitted.

Despite its limitations, our study is one of the first to describe hospital‐related COVID‐19 outcomes, readmission rates with causes, and resource utilization in patients with cirrhosis in the U.S. population at a national level. Our findings indicate that COVID‐19 infection is associated with an increased risk of all‐cause inpatient mortality and resource utilization in patients with cirrhosis. Moreover, a considerable number of cirrhosis patients get readmitted with sepsis post COVID infection for reasons that are currently not understood. This leads to increased resource utilization, adding a burden to the healthcare costs at the national level. Our study results should encourage future studies to address the causes of these readmissions to improve morbidity and mortality in these patients. This study also adds to the growing body of evidence for encouraging mandatory immunization and booster doses in patients with cirrhosis. Because of their high risk of developing severe disease, early initiation of advanced COVID‐19 therapies during hospitalization could improve the outcomes for these patients. Although this data predates the availability of vaccines and advanced treatments for COVID‐19, it would be interesting to see the results of future studies to see whether these trends persist for subsequent years of the pandemic.

## Supporting information


**Data S1.** Supplementary file with ICD‐10 codes.
