# Supplementary material for: Predictors of inpatient outcomes of COVID‐19 infection in patients with cirrhosis in the early pandemic phase: A nationwide survey
Source: JGH Open. 2023 Nov 8;7(12):889–98. doi: 10.1002/jgh3.12998 (PMC10757488; doi:10.1002/jgh3.12998)
Supplement: Supplementary file 1 — Data S1. Supplementary file with ICD‐10 codes. [file JGH3-7-889-s001.docx]

Supplementary File with ICD-10 Codes

| Condition | ICD-10 Codes |
| --- | --- |
| COVID-19 | U071 |
| Cirrhosis | K743, K744, K745, K7469, K7460, K740, K7030, K7031, K702, K742, K7151, K717 |
| Portal Hypertension | K766 |
| Ascites | R188, K7031, K7011, K7151 |
| Varices | I8511, I8501 |
| Hepatic Encephalopathy | G9340, G9341, G9349, R40, K7041, K7111, K7201, K7211, K7291, B190, B1911, B1921 |
| Acute Respiratory Failure | J9600, J9690, J80, J95821, J952, J953, J95811, J95812 |
| Mechanical Ventilation | 5A1955Z, 5A1945Z, 5A1935Z |
| Vasopressors | 3E030XZ, 3E033XZ, 3E040XZ, 3E043XZ, 3E050XZ, 3E053XZ, 3E060XZ, 3E063XZ |
| Septic Shock | R65.21 |
| Hypertension | I10 |
| Diabetes Mellitus | E11 |
| Acute Kidney Injury | N17 |
| Chronic Kidney Disease | N18 |
| Stage 2 | N18.2 |
| Stage 3 | N18.3 |
| Stage 4 | N18.4 |
| Stage 5 | N18.5 |
| Unspecified | N18.9 |
| ESRD | Z992, N186 |
| Chronic Pulmonary Disease | J40-J47 |
| Congestive Heart Failure | I50 |
